# Dr. Parker’s Clinique; College of Physicians and Surgeons. March 5, 1849

**Published:** 1849-04

**Authors:** 


*Dr. Parker's Clinique; College of Physicians and Surgeons. March* 5, 1849.

*Case* 1*st.*—Boy, aged 7 years, with strumous or scrofulous conjunctivitis. Dr. Parker remarked that this case was important as illustrating a particular form of ophthalmia—one, too, in which accuracy of diagnosis is all important, in view of successful treatment. Indeed, remarked the doctor, we have with the eye, as with the ear, too little careful discrimination in regard to the different forms of disease which affect its various structures. If called to a patient with redness, heat, and pain in the eye, the physician is too apt to pronounce it *ophthalmia*, and make out his prescription, without the least inquiry into the question, whether the inflammation is located in the conjunctiva, the sclerotica, the iris, or any other of the structures of the eye. And as little do they inquire whether it is catarrhal, scrofulous, rheumatic, or syphilitic in its character. On this account many blunders are made in the treatment, and many eyes irretrievably lost. In the case before us, we have an enlarged and congested state of the vessels of the conjuctiva, forming a net-work over the whole surface of the eye-ball except the cornea. There is some intolerance of light, and the pain is of that smarting, rough character which likens it to the pain arising from sand or dirt in the eye. The lids are agglutinated together in the morning, showing the existence of lipitudo or disease of the Meibomian glands. There are also some small pustules, near the base of the cornea, which if allowed to go on, would, doubtless, lead to small irritable ulcers. The disease in this case has continued eight or ten days, and affects both eyes. It is of a sub-acute character. From inflammation of the sclerotic coat, it is distinguished by the character of the redness and pain. The vessels in sclerotitis, instead of showing a prominent net-work, as in this case, seem to radiate in lines from the base or edge of the cornea, as from a centre, are less prominent, and more nearly of a brick-red color. The pain in the eye, is deeper seated, and like rheumatism, most severe at night. From simple catarrhal conjunctivitis, it is distinguished by the general scrofulous aspect of the patient, by the greater intolerance of light, and especially by the pustules already alluded to. These latter are characteristic of the scrofulous ophthalmia.

*Treatment.*—This must depend entirely on the stage and character of the inflammation. In this case which is sub-acute, of several days continuance, and presenting evident marks of scrofula, the treatment should be mainly constitutional; 4 grs. of calomel, or 6 grs. of pilulæ hydrarg., given at night and followed in the morning, by rhei and soda, sufficient to operate moderately on the bowels, followed in two or three days by small doses of the iodide of iron, or of bi-chloride of mercury dissolved in tincture of cinchona, would, doubtless, speedily effect a cure. Locally, a collyrium, composed of

℞. Rose Water.... ℥i.
Sulph. Zinc.....4 grs.
Vinum Opii....30 gtts.—Misce.

dropped into the eye two or three times a day, and an ointment composed of Unguentum Hydrargyri nitratis, largely diluted with rose ointment, applied to the edge of the eye-lids every night will generally much facilitate the recovery. In cases of a more acute character, local bleeding may be necessary in the commencement.

The strength of all local applications to the eye should be regulated by their effects. The smarting induced by them, should never exceed five minutes in duration. No *poultices* should ever be applied to an inflamed eye.—*Annalist.*

